# Using Artificial Intelligence and Novel Polynomials to Predict Subjective Refraction

**DOI:** 10.1038/s41598-020-65417-y

**Published:** 2020-05-22

**Authors:** Radhika Rampat, Guillaume Debellemanière, Jacques Malet, Damien Gatinel

**Affiliations:** Foundation Adolphe de Rothschild Hospital, Paris, France

**Keywords:** Imaging and sensing, Machine learning

## Abstract

This work aimed to use artificial intelligence to predict subjective refraction from wavefront aberrometry data processed with a novel polynomial decomposition basis. Subjective refraction was converted to power vectors (M, J0, J45). Three gradient boosted trees (XGBoost) algorithms were trained to predict each power vector using data from 3729 eyes. The model was validated by predicting subjective refraction power vectors of 350 other eyes, unknown to the model. The machine learning models were significantly better than the paraxial matching method for producing a spectacle correction, resulting in a mean absolute error of 0.301 ± 0.252 Diopters (D) for the M vector, 0.120 ± 0.094 D for the J0 vector and 0.094 ± 0.084 D for the J45 vector. Our results suggest that subjective refraction can be accurately and precisely predicted from novel polynomial wavefront data using machine learning algorithms. We anticipate that the combination of machine learning and aberrometry based on this novel wavefront decomposition basis will aid the development of refined algorithms which could become a new gold standard to predict refraction objectively.

## Introduction

Globally it is estimated that 153 million people aged 5 or above are visually impaired due to uncorrected refractive errors^1^.The ability to automatically refract a patient and provide a spectacle prescription, equivalent to the time consuming current gold standard of subjective refraction, is an elusive goal that has intrigued many ophthalmic clinicians and researchers^2–4^.

One such automated and objective method is optical wavefront sensing using aberrometry which allows mathematical reconstruction and analysis of lower and higher order monochromatic aberrations of the eye. This has led many to believe that this objective method had the potential to be the new standard for optimizing correction of refractive errors by converting aberrometry data to accurate sphero-cylindrical refractions^5–7^.

Though several small sample studies showed promising results in terms of accuracy and precision of objective refraction from several methods related to wavefront analysis to date^5–13^ no study has found a validated method that can be used to prescribe a spectacle correction. It was found that results from the aberrometer, autorefractor and subjective refraction, though comparable with each other, were not accurate enough to prescribe spectacles directly from either instruments^14^. A recent publication found that a visual image quality metric could predict subjective refraction in myopic eyes but not habitually undercorrected hyperopic eyes, though the data set was again small^15^. Variability in the gold standard subjective refraction measurements themselves were also thought to be a source of poor precision^7^.

The ocular wavefront error is most commonly described by the Zernike polynomials^16^. To satisfy orthogonality constraints with low order modes, some higher order Zernike polynomials contain low order terms in their analytical expression leading to lack of accuracy when predicting the sphero-cylindrical refraction^17^. It is known that conventional therapies such as spectacles or contact lenses correct just the lower-order aberrations but the presence of higher-order aberrations influences the prescription itself^12^. An important finding by Cheng *et al*.^6^ showed that subjective judgment of best focus does not minimize RMS wavefront error (Zernike defocus = 0), nor create paraxial focus (Seidel defocus = 0), but makes the retina conjugate to a plane between these two. The levels of spherical aberration (Z_4_°) and secondary astigmatism (Z_4_^±2^) influenced the levels of defocus and primary astigmatism that produced the best visual performance. These objective metrics were tested based on an assumption that the Zernike polynomial decomposition was producing a clear distinction between the low and high order components of the wavefront error. This assumption could explain the poor correlation between subjective and objective refraction, especially when it came to large amounts of higher order aberration^5–7,9,11,12,18,19^.

A new series of polynomials, labeled LD/HD (Low Degree/ High Degree), have been proposed to provide a more mathematically relevant separation of the higher and lower modes^20^. In this decomposition, the normalized higher order modes are devoid of low order terms and mutually orthogonal within but not with lower order aberrations. With this approach, the low order wavefront component is equal to the paraxial curvature matching of the wavefront map.

Machine learning is already in use in Ophthalmology for image analysis in medical retina^21–23^, and glaucoma (Visual Fields and Disc Photos)^24,25^ as well as recent developments for use in diagnoses including retinopathy of prematurity^26^. It is also used in regression tasks, notably in IOL calculations^27^. Deep learning has been applied to predict refractive error from fundus images and other image analysis techniques also^28,29^. Attempts to predict subjective refraction from Zernike polynomials have also been tried using a multilayer perceptron with two hidden layers^30^.

Our aim was to build and evaluate a set of predictive machine learning models to accurately and precisely objectively refract a patient using wavefront aberrometry with LD/HD polynomial decomposition, and to evaluate the relative importance of each polynomial in the prediction process for each vector.

## Results

### Group comparability

Patients demographics are presented in Table 1. Training set and test set were comparable in terms of patient ages, sex-ratio, side repartition, mean refractive spherical equivalent and mean refractive cylinder.

**Table 1:** Patients demographics in the training set and test set. T-test was performed to test for group comparability. Abbreviations used: Spherical Equivalent (SE), Cylinder (Cyl) and number (n). SE and Cyl are in Diopters (D).

	Training set				Test set				p value
n eyes	3729				350				—
n patients	1809				193				—
Female %	57.1%				58.9%				0.71
Right Eye %	49.7%				49.4%				1.00
	**Mean**	**SD**	**Min**.	**Max**.	**Mean**	**SD**	**Min**.	**Max**.	
Age	36.03	11.24	18.10	72.40	36.35	11.48	20.00	66.00	0.71
Refractive SE	−1.89	2.54	−6.75	6.13	−1.96	2.58	−6.63	4.88	1.00
Refractive Cyl	−0.81	0.90	−6.00	0.00	−0.87	0.94	−5.25	0.00	1.00

### Prediction performance of the different methods

**Figure 1:**
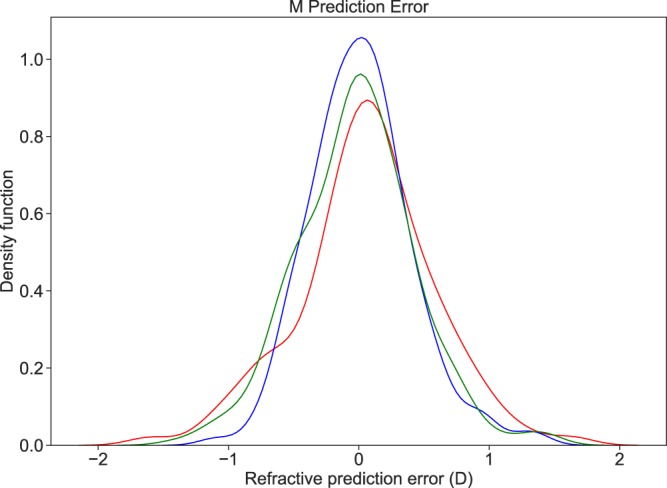
Probability density function (Gaussian kernel density estimate) for the Spherical Prediction Error, for the 3 methods studied. We compare paraxial fitting with low degree LD/HD polynomials (Red), with XGBoost model using low degree only (Green) and XGBoost model with all aberrations (Blue). The density of accurate predictions is more important with the latter.

**Figure 2:**
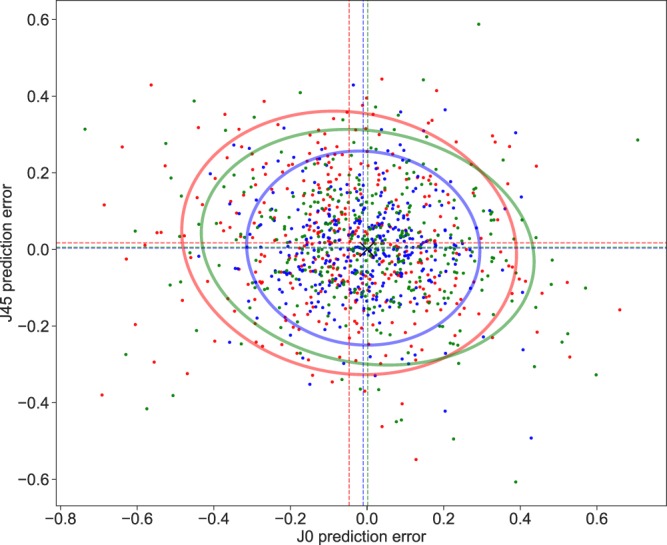
Scatter plot showing the J0 vector prediction error on the X-axis and J45 vector prediction error on the Y-axis with corresponding 95% confidence ellipses for the 3 methods studied. We compare paraxial fitting with low degree LD/HD polynomials (Red), with XGBoost model using low degree only (Green) and XGBoost model with all aberrations (Blue). The black cross locates the (0,0) coordinate. Precision is better using the last method.

The performance of prediction methods are presented in Table 2. Figure 1 illustrates the prediction performances of the different approaches for the M vector and Fig. 2 illustrates those for the J0 and J45 vectors. Statistical testing for differences between the various prediction methods is presented in Table 3. The XGBoost models using all the polynomials, resulted in a Mean Absolute Error of 0.30 ± 0.25 Diopters (D) for the M vector, 0.12 ± 0.09 D for the J0 vector and 0.09 ± 0.08 D for the J45 vector, whilst the Paraxial matching method resulted in a Mean Absolute Error of 0.40 ± 0.35 D for the M vector, 0.17 ± 0.14 D for the J0 vector and 0.14 ± 0.1 D for the J45 vector. The XGBoost models using only the low-degree polynomials resulted in a Mean Absolute Error of 0.35 ± 0.29 D for the M vector, 0.16 ± 0.14 D for the J0 vector and 0.12 ± 0.10 D for the J45 vector. Bland-Altman plots showed a good agreement between subjective refraction and the predictions obtained with the machine learning models, with no systematic error depending on degree of refractive error (Fig. 4). Paired t-test were not significant.

**Table 2:** Absolute Prediction Error, Prediction Error and Precision of the three methods were evaluated (Paraxial Matching, XGBoost using Low order polynomials only, XGBoost using all available polynomials) for each vector M, J0 and J45. P values for the comparison between methods are presented in Table 3. Abbreviations used: Low Order (LO), Low and High Order (LO/HO) and Paraxial Matching (PM).

Prediction Method	Absolute Prediction Error				Prediction Error (Accuracy)				Precision
	Mean	SD	Min.	Max.	Mean	SD	Min.	Max.	
PM -M	0.40	0.35	0.00	1.66	0.06	0.53	−1.66	1.66	1.06
XGB (LO only) -M	0.35	0.29	0.00	1.50	-0.02	0.46	−1.43	1.50	0.91
XGB (LO/HO) -M	0.30	0.25	0.00	1.41	0.01	0.39	−1.14	1.41	0.78
PM - J0	0.17	0.14	0.00	0.69	-0.05	0.22	−0.69	0.66	0.44
XGB (LO only) - J0	0.16	0.14	0.00	0.74	0.00	0.22	−0.74	0.71	0.43
XGB (LO/HO)- J0	0.12	0.09	0.00	0.44	-0.01	0.15	−0.44	0.43	0.30
PM - J45	0.14	0.10	0.00	0.55	0.02	0.17	−0.55	0.45	0.34
XGB (LO only)- J45	0.12	0.10	0.00	0.61	0.01	0.15	−0.61	0.59	0.31
XGB (LO/HO)-J45	0.09	0.08	0.00	0.49	0.00	0.13	−0.49	0.43	0.25

**Table 3:** Pairwise statistical comparison of the different prediction methods. Mean Absolute Errors and Mean Errors were compared using a Wilcoxon signed-rank test and Precision differences were compared using the Levene test for equal variances. Abbreviations used: Low (L), High (H), Paraxial Matching (PM).

Prediction Method	Mean Absolute Error	Mean Error (Accuracy)	Precision
**M vector prediction**			
XGB L & H/PM	p < 0.0001	p < 0.001	p < 0.0001
XGB L/PM	p < 0.0001	p < 0.0001	p = 0.06 (NS)
XGB L/XGB L & H	p < 0.0001	p < 0.001	p = 0.02 (NS)
**J0 vector prediction**			
XGB L & H/PM	p < 0.0001	p < 0.0001	p < 0.0001
XGB L/PM	p = 0.05 (NS)	p < 0.0001	p = 0.82 (NS)
XGB L/XGB L & H	p < 0.0001	p = 0.03 (NS)	p < 0.0001
**J45 vector prediction**			
XGB L & H/PM	p < 0.0001	p = 0.03 (NS)	p < 0.0001
XGB L/PM	p < 0.0001	p = 0.03 (NS)	p = 0.003
XGB L/XGB L & H	p < 0.0001	p = 0.48 (NS)	p = 0.003

### Pairwise prediction methods comparison

The XGBoost models using all the polynomials performed statistically better than Paraxial matching for every vector and every metric, except for accuracy for the J45 vector prediction. They also performed better than the XGBoost models trained with low-degree polynomials only, although the difference was not significant for precision in predicting the M vector and accuracy in predicting the J0 and J45 vectors.

### Feature importance

SHAP value analysis for the three XGBoost models trained with the full set of polynomials is presented in Fig. 4. It showed that G_2_^0^(defocus) was by far the most influential feature to predict the M vector, with G_4_° (primary spherical aberration) being the second most important feature. The bottom two graphs demonstrate that G_2_^2^ (Vertical astigmatism) and G_4_^−2^ (Oblique secondary astigmatism) were the most important features to predict the J0 vector, while G_2_^−2^ (Oblique astigmatism) and G_4_^2^ (Vertical secondary astigmatism) were the most important features to predict the J45 vector.

## Discussion

The machine learning approach using LD/HD polynomials was more effective than the paraxial matching method for predicting the results of conventional, sphero-cylindrical refraction from wavefront aberrations used by Thibos et al. previously^7^. Interestingly, the XGBoost models trained using low-order aberrations only proved more accurate than paraxial matching. This could suggest that those low-order polynomials interact, in some circumstances, in a more complex way than previously thought. The best precision and accuracy were obtained when all the novel polynomials coefficients were used as predictors, demonstrating the significant influence of the higher order aberrations on the spectacle correction.

Gradient boosting creates new models that predict the residual errors of prior models during the training process. The models are used together to predict the target value. XGBoost is an implementation of gradient boosted trees focused on performance and computer efficiency. It can perform both regression and classification tasks. It was chosen because of its recognized performances and its resistance to overfitting^31^.

Feature importance G_2_° (defocus) was unsurprisingly the most influential feature to predict the M vector, with G_4_° (primary spherical aberration) being the second most important feature. One interesting finding was that G_4_^−2^ (Oblique secondary astigmatism) was the second most important feature to predict J0, and G_4_^2^ (Vertical secondary astigmatism) the second most important feature to predict J45, while the inverse would be more intuitive. This demonstrates the interest in the machine learning approach, that allows us to discover new patterns and relationships between predictors by disregarding previous assumptions.

Our results confirm the prevalence of 4th order aberrations within the higher order coefficients influencing the sphero-cylindrical refraction as it has been previously shown^6^. The LD/HD modes being devoid of defocus terms (radial degree 2), they unambiguously confirm the influence of the radial degree 4 of the wavefront error, on sphero-cylindrical refraction.

The predictive influence of the variables used in the model does not explain their exact role, and that is a weakness of such machine learning algorithms, as interpretability and model comprehension are limited by the big number of decision trees, their complexity and depth.

Of note, we did not test our method for repeatability. However, it relies solely on the OPD-Scan III output, and this device has already shown very good repeatability^32–35^.

Our study had some unavoidable limitations, among which is accommodation. We created a study design using undilated refraction, mirroring the real life clinical environment where spectacle correction is provided in adults, as well as allowing preservation of data volume. We did not test children or elderly patients so cannot generalize to these groups. By virtue of the technique, it is not possible to objectively refract patients with strabismus, corneal scarring, cataracts or vitreous opacity that would preclude clear wavefront analysis.

Precision may be masked by the imprecision of the gold standard of subjective refraction. Of note the examiner was aware of the autorefraction. We hope our study results will enable future development of machine learning algorithms from the LD/HD polynomials and objective refraction techniques, to prescribe glasses efficiently, not only to adults but also to children and vulnerable adults without need for their input or prolonged cooperation.

## Methods

### Patients and dataset constitution

This study was approved by the Institutional Review Board at Rothschild foundation and followed the tenets of the Declaration of Helsinki. Informed consent was obtained from all participants. A total of 2890 electronic medical records of patients (6397 eyes) evaluated for refractive surgery at the Laser Vision Institute Noémie de Rothschild (Foundation Adolphe de Rothschild Hospital, Paris) were retrieved and consenting patients data was analyzed. We excluded patients with strabismus and any other ocular abnormalities except ametropia. After data cleaning, eyes with subjective refraction and a valid wavefront aberrometry examination were randomly split into a 350 eyes test set and a training set, with no cross over of same patient data. A manual review of medical records of eyes in the test set was checked to ensure the quality of data, leaving 3729 eyes for the training set.

### Aberrometry

Wavefront analysis was obtained using the OPD-Scan III (Nidek, Gamagori, Japan). The aberrometer was specially configured to run using beta-software incorporating the new series of LD/HD polynomials, noted G_n_^m^ using the same double index scheme of the Zernike polynomials. The wavefronts were decomposed up to the 6^th^ order. We chose to stop our polynomials analysis at the 6^th^ order. This cut-off is beyond the number of polynomials that was determined by the members of the Vision Science and its Applications Standards task force (VSIA) to be necessary to describe the HOA of the human eye with sufficient accuracy in 2000^36^. It applied to the paraxial matching analysis as well as the machine learning approach. The first three polynomials (Piston, Tilt, Tip) were removed from the features because of their low relevance in this work. Defocus, Vertical Astigmatism and Oblique Astigmatism constituted the Low order polynomial group, and all the others constituted the High order polynomials group. A 4 mm pupil disk diameter was chosen to obtain the coefficients and any pupil less than 4 mm during the acquisition of the wavefront with the OPD-Scan III, was an exclusion criterion. A 4 mm pupil diameter analysis cut-off was used because it is close to the mean physiological photopic pupil diameter in different studies^37–39^. Our results may not reflect the results that could be found using very large or very small pupils.

### Subjective refraction

Corresponding non-cycloplegic subjective refractions conducted on the same day by an experienced optometrist were analyzed. The maximum plus rule was used to the nearest 0.25 D to minimize accommodation and maximize the depth of focus^7^.

### Power vector analysis

Each refraction in Sphere S, Cylinder C, and axis A format was transformed into 3D dioptric vector space (M, J0, J45) where the three components are orthogonal. Refraction data sets were vectorized using standard power vector analysis with the components M, J0 and J45^40^ using Eqs. (1), (2) and (3).

1 $${\rm{M}}=S+\frac{C}{2}$$

2 $${\rm{J0}}=-\,\frac{C}{2}\times \,\cos (2\alpha )$$

3 $${\rm{J45}}=-\,\frac{C}{2}\times \,\sin (2\alpha )$$

### Machine learning methodology

Three machine learning models were separately trained to predict each vector component from the new series of polynomials. We used a Gradient Boosted Trees algorithm (XGBoost)^41^. Parameter tuning was performed using 5-folds randomized search cross-validation. Mean squared error regression loss was chosen as the evaluation metric. We used Python 3.6.8 with the following libraries: Jupyter 4.4.0. Pandas 0.23.4, Scikit-learn 0.20.2, Matplotlib 3.0.2, Seaborn 0.9.0, XGBoost 0.81.

### Feature importance analysis

**Figure 3:**
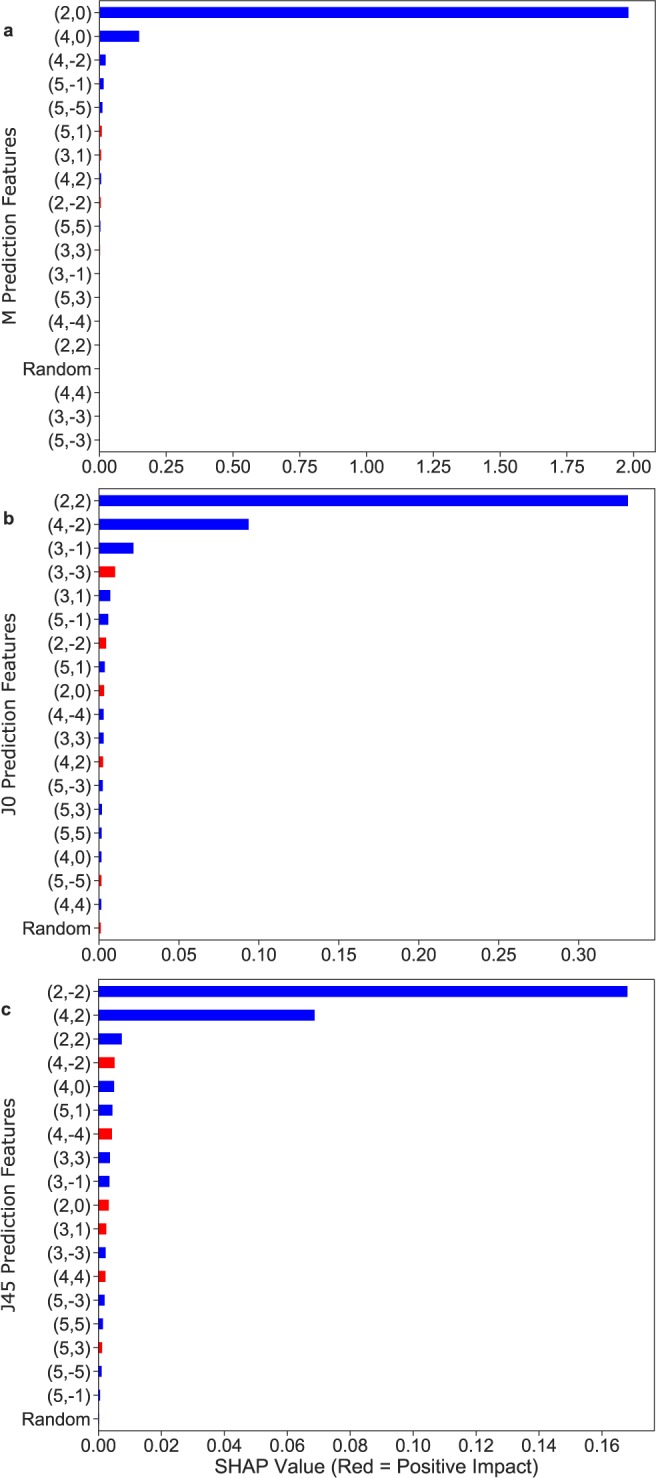
SHAP feature importance for each model of the XGBoost using the all aberrations approach. The top graph (**a**) displays the most important features for M prediction: G_2_° (defocus) and G_4_° (primary spherical aberration) were the most influential. The bottom two (**b**,**c**) graphs demonstrate that G_2_^2^ (Vertical astigmatism) and G_4_^−2^ (Oblique secondary astigmatism) were the most important features to predict the J0 vector, while G_2_^−2^ (Oblique astigmatism) and G_4_^2^ (Vertical secondary astigmatism) were the most important features to predict the J45 vector.

SHAP (SHapley Additive exPlanations) values were calculated for each model in order to determine the most influential polynomials (Fig. 3).

**Figure 4:**
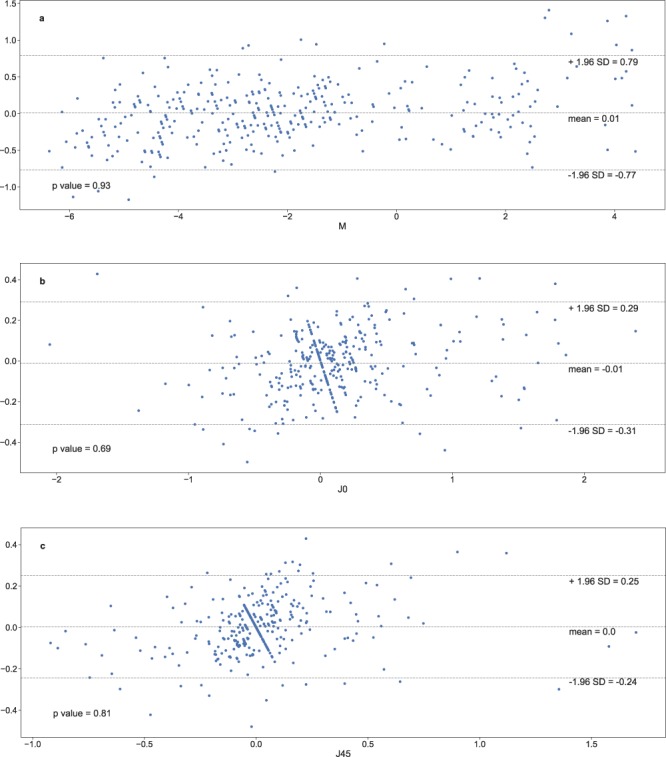
Bland-Altman diagrams showing the agreement between the predictions made using the XGBoost model trained with all available aberrations, and subjective refraction, for M (**a**), J0 (**b**) and J45 (**c**). No statistical difference was found using one sample t-test, for each vector prediction.

SHAP value is a recently described tool that aims to increase machine learning models interpretability^42^. It allows us to understand how a specific feature negatively or positively participates in the target variable prediction by computing the contribution of each feature towards the prediction. This allows better estimation of the importance of each feature in the prediction process. A random variable was introduced as a predictive feature during the training in order to help differentiate useful features from the others.

### Model evaluation and statistical analysis

Performances of the machine learning models were evaluated on the test set never seen by the model nor used for the hyperparameters choice, to avoid overfitting. For each machine learning approach (using low order polynomials only, and using every polynomial), the three vectors of the refraction were predicted one by one using the three machine learning models. Paraxial matching predictions were calculated using Eqs. (4–6)

4 $${\rm{M}}=\frac{-{G}_{2}^{0}4\sqrt{3}}{{r}^{2}}$$

5 $${\rm{J}}0=\frac{-{G}_{2}^{2}2\sqrt{6}}{{r}^{2}}$$

6 $${\rm{J45}}=\frac{-{G}_{2}^{-2}2\sqrt{6}}{{r}^{2}}$$

where G_n_^m^ is the *n*^th^ order LD/HD coefficient of meridional frequency *m*, and r is the pupillary radius. It is important to note that as high order LD/HD coefficients are devoid of low-order aberrations, this calculation is equivalent to paraxial curvature matching calculated by computing the curvature at the origin of the Zernike expansion of the Seidel formulae for defocus and astigmatism using Zernike polynomials as described by Thibos *et al*.^7^.

Mean absolute errors were calculated for each prediction method. Accuracy of the predictions for each vector was defined as the mean value of the prediction error. Precision was defined as two times the standard deviation (SD) of the prediction error^7^. Each prediction method was evaluated against each other. Mean absolute prediction errors and mean prediction errors were evaluated using the Wilcoxon-signed rank test with Bonferroni correction. Differences in precision were evaluated using the Levene test with Bonferroni correction. We used a similar confidence ellipse to Thibos et al. to graphically report our results^7,43^. Bland-Altman plots and paired t-test were conducted to study the agreement between subjective refraction and the machine learning models predictions. A p-value less than 0.05 was considered significant.

## Data Availability

The datasets generated during and analyzed during the current study are available from the corresponding author on reasonable request.
